# Effects of delayed hip replacement on postoperative hip function and quality of life in elderly patients with femoral neck fracture

**DOI:** 10.1186/s12891-020-03521-w

**Published:** 2020-07-24

**Authors:** Jidong Song, Gensheng Zhang, Jialin Liang, Chuanyi Bai, Xiaoqian Dang, Kunzheng Wang, Caiyou He, Ruiyu Liu

**Affiliations:** 1 Department of Orthopaedics, the Second Affiliated Hospital of Xi’an Jiaotong University, Xi’an Jiaotong University, NO.157, Xiwu Road, Xi’an, Shaanxi 710004 People’s Republic of China; 2 3201 Hospital of Xi’an Jiaotong University Health Science Center, Xi’an Jiaotong University, Hanzhong, Shaanxi Province 723000 People’s Republic of China

**Keywords:** Delayed femoral neck fracture, Hip replacement, Function, Quality of life

## Abstract

**Background:**

For various reasons, some elderly patients with femoral neck fracture undergo delayed surgical treatment. There is little information about the effect of delayed treatment on postoperative hip function and quality of life. The aim of this study was to investigate the effect of delayed hip arthroplasty on hip function, quality of life, and satisfaction in patients with femoral neck fractures.

**Methods:**

Forty-seven patients with femoral neck fracture and hip replacement delayed over 21 days served as the delayed group (D group). Patients with femoral neck fracture, matched 1:1 for age and sex, and hip replacement within 7 days served as the control group (C group). The Harris hip score (HHS) and health-related quality of life (HRQoL) were assessed before surgery and 3 months, 6 months and 1 year postoperatively. The satisfaction questionnaires were completed by the patients themselves at the last follow-up.

**Results:**

The HHS in the C group was lower than that in the D group (32.64 ± 9.11 vs. 46.32 ± 9.88, *P* < 0.05) before surgery but recovered faster after surgery. The HHS in the D group was lower than that in the C group 1 year postoperatively (85.2 ± 3.80 vs. 89.8 ± 3.33, *P* < 0.05). The patients’ quality of life changed similarly to their HHS. The HHS 1 year after surgery was related to the preoperative HHS in group D (r_s_ = 0.521, *P* < 0.01). Patients in the D group showed significantly higher satisfaction scores than those in the C group (*P* < 0.05).

**Conclusions:**

Hip function in patients with femoral neck fracture surgery delayed over 21 days recovered more slowly than that in those who underwent surgery within 7 days. However, they were more satisfied with the surgery. Moderate hip movement to ameliorate the lower limb muscle atrophy was recommended for patients facing a temporary inability to undergo surgery.

## Background

Femoral neck fracture is one of the most common and serious fractures in elderly patients [1]. Globally, the estimated number of femoral neck fractures is set to peak at 6.3 million by 2050 [2]. Although femoral neck fracture represents only 14% of all osteoporotic fractures, it accounts for 72% of fracture-related medical expenses [3]. It has understandably been a major public health problem owing to high morbidity, mortality, and healthcare expenses. Many evidence-based guidelines recommend surgical interventions at the earliest possible time for elderly patients with femoral neck fractures with the aim of reducing postoperative complications [4, 5]. However, some surgeries may be delayed because of various factors, such as the need to optimize medical comorbidities, patient choice, misdiagnosis or a missed diagnosis [6, 7].

There is a wealth of research on the effects of surgical delay on mortality, length of hospital stay, and postoperative complications in elderly patients with femoral neck fracture [8–10]. Only a limited number of studies have examined the relationship between surgical delay and postoperative hip function and quality of life, and they found inconsistent results [11, 12]. A long surgical delay was associated with poor function and early surgical treatment improved patients’ ability to return to independent living [11, 13]. In contrast, Orosz et al. reported that early surgery was not associated with improved function but rather with reduced pain [9]. These studies typically focused on a delay of 24 to 48 h. We often encounter elderly patients with femoral neck fracture whose delay is longer than 48 h, sometimes much longer. A long waiting time for surgery is associated with delayed mobilization rehabilitation and increased pain [14, 15]. Prolonged immobility may result in muscle disuse of the affected hip and delayed postoperative function recovery, eventually leading to a decline in quality of life. However, there are few data concerning the recovery of hip function and quality of life of patients following delayed femoral neck fracture surgeries and how to improve this condition, which is essential for the guidance of rehabilitation for these patients.

This 1-year prospective study aimed to investigate the effect of delayed hip replacement on hip function and quality of life in elderly patients with femoral neck fracture and the causes of delay to ascertain which factor was associated with postoperative function. The analysis of causes of such delays and the in-depth study of the efficacy of long-delayed surgical treatment in patients with femoral neck fractures will help improve the reality of delayed surgical treatment for femoral neck fractures in developing countries and benefit future patients undergoing such treatment.

## Methods

This study was undertaken in our hospital between February 2014 and August 2018 and was approved by the ethics committee of the hospital (No. 2013–127).

The patient inclusion criteria were as follows: (a) age > 65 years; (b) hip replacement> 21 or < 7 days after unilateral femoral neck fracture; (c) ability to provide informed consent. The patient exclusion criteria were as follows: (a) pathological or other fractures; (b) previous lower extremity surgery; and (c) neurological, muscular, or congenital low extremity diseases or other joint diseases. Patients undergoing hip replacement> 21 days after fracture were matched 1:1 for age, sex and surgery methods with those undergoing surgeries< 7 days after fracture.

Many elderly patients with femoral neck fracture have various comorbidities, such as hypertension, diabetes, cardiovascular diseases and lung disease. Most of these diseases can be improved within 7 days and surgery can be performed with a low risk. These patients were regarded as the control group (C group). Some patients with femoral neck fracture have a long-delayed surgical treatment due to fear of the high surgical risk resulting from severe cardiopulmonary comorbidities, preference for nonoperative treatment, or other causes. Femoral neck fractures which surgical treatment was delayed over 21 days were regarded as old fractures [16, 17]. Therefore, patients with old femoral neck fractures were regarded as the delayed group (D group).

All operations were performed by the same team of surgeons. Prophylactic antibiotics were given half an hour before the surgery. Patients were given general anesthesia or epidural anesthesia and placed in the lateral position. The posterolateral approach to the hip joint was used. The operation method and implant selection were determined according to patient age and general physical status. In general, total hip replacement was performed in patients under the age of 70 or who were in good physical condition, while femoral head replacement was performed in patients over 70 years old or who were in poor physical condition. For patients with delayed surgery, the hip capsule was partly resected, and the iliac psoas muscle was released if it was difficult to render hip prosthesis reduction during the surgery. After equipment and reduction of the hip prosthesis, the tension of the hip adductor was tested by abducting the hip joint. The adductor muscle tendon was partly resected in the inner thigh to reduce overly high adductor tension, which can limit hip abduction and is apt to result in postoperative hip dislocation. After surgery anticoagulant therapy was performed to prevent deep vein thrombosis for 5 weeks if the patients had no contraindications for anticoagulation.

The Harris hip score (HHS) and health-related quality of life (HRQoL) were assessed by trained interviewers one day before surgery and 3 months, 6 months and 1 year postoperatively. Satisfaction questionnaires were completed by patients at the last follow-up. The HHS is an outcome tool typically used following total hip replacements [18]. It is now also used for the assessment of femoral neck fractures [19]. The HHS comprises 10 items grouped into four domains: pain (1 item, 0–44 points); function (7 items, 0–47 points); absence of deformity (1 item, 4 points); and range of motion (2 items, 5 points). The score has a maximum of 100 points, and higher scores indicate less dysfunction and better outcomes.

HRQoL was assessed using the EQ-5D score [20]. The EQ-5D has five dimensions: mobility, self-care, usual activities, pain/discomfort, and anxiety/depression. Each dimension is divided into three degrees of severity: no problem, some problems, and major problems. Then, the health index score was calculated using the Japan population-based time-off models [21]. Higher scores indicate better HRQoL.

Patient satisfaction was investigated using a self-administered patient satisfaction scale at the last follow-up [22]. Four items including overall satisfaction, pain relief, the ability to perform home or yard work, and the ability to perform recreational activities, were graded on a 4-point Likert scale ranging from 25 to 100 per item (100 indicates most satisfied). The final score is the unweighted mean of scores from the 4 items.

Comorbidities, on the basis of Charlson’s comorbidity index [23], and causes of delay for each patient were assessed preoperatively through history and examination. Postoperative complications were observed, including nerve injury, infection and venous thromboembolism. Postoperative dislocation and prosthetic loosening were evaluated by hip radiography.

Statistical tests were performed using SPSS version 20 (SPSS Inc., Chicago, Illinois). The results are presented as the mean ± *SD*. The Chi-square test was used to analyze differences between proportions. Independent-Samples *t*-tests were used to compare continuous variables between groups at each time point. Spearman’s correlation was used to analyze the relationship between the preoperative HHS and the HHS 1 year postoperatively in the D group. Simple linear regression was used to analyze the effect of time on the HHS. *P* < 0.05 was considered statistically significant.

## Results

### Participant characteristics

Forty-seven patients with unilateral femoral neck fracture who underwent hip replacement after a 21-day delay after fracture were included in the D group. Forty-seven patients, matched 1:1 for age, sex, ASA scores and surgery methods, who had surgeries within 7 days after fracture were in the C groups. Their baseline characteristics are listed in Table 1. The mean age was 70.6 ± 8.1 years and 73.0 ± 7.0 years in the C and D groups, respectively. The average time from fracture to surgery in the C group was 2.9 ± 1.8 days, while that in the D group was 115.1 ± 51.1 days.

**Table 1:** Baseline characteristics of C and D group

	C group	D group	*P* value ^a^
**Patients (number)**	47	47	–
**Demographics**			
Age (years)	70.6 ± 8.1	73.0 ± 7.0	0.124
Female (%)	36.0	34.0	0.829
BMI (body mass index: kg/m^2^)	21.68 ± 2.46	21.88 ± 1.78	0.655
Surgical delay time (days)	2.9 ± 1.8	115.1 ± 51.1	<0.001
**Surgery methods (number)**			
Femoral head replacement	27 (57.4%)	31 (66.0%)	0.396
Total hip replacement	20 (42.6%)	16 (34.0%)	
**Charlson’s comorbidities index**	3.1 ± 1.1	3.3 ± 1.0	0.352

^a^ Independent-Samples *t*-test was used for analyzing differences between means. The Chi-square test was used for analyzing differences between proportions

### Functional outcomes

**Fig. 1:**
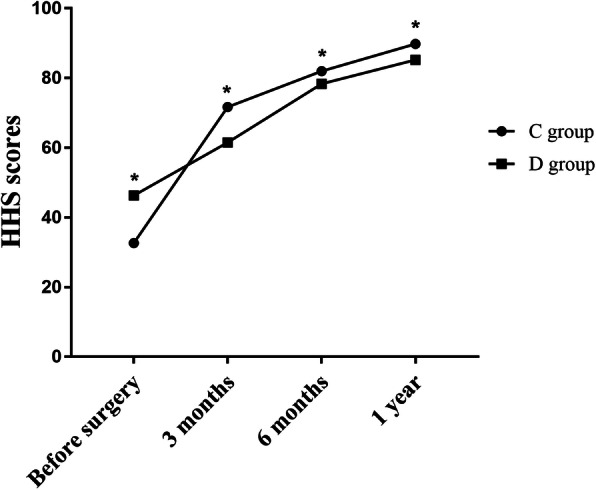
HHS scores before surgery and at 3 months, 6 months and 1 year postoperatively in both groups * indicates *P*<0.05

Two patients in group C were lost to follow-up by 3 months postoperatively and one patient in group D died of myocardial infarction 2 months postoperatively. For the remaining 45 and 46 patients in the C and D groups, respectively. HHSs were available before surgery and at 3 months, 6 months and 1 year postoperatively (Table 2, Fig. 1).

**Table 2:** Mean HHS scores before surgery and 3 months, 6 months and 1 year postoperatively in both groups

	C group	D group	*P* value
Before surgery	32.64 ± 9.11	46.32 ± 9.88	<0.001
3 months postoperatively	71.71 ± 4.78	63.02 ± 6.17	<0.001
6 months postoperatively	81.96 ± 4.94	78.35 ± 3.56	<0.001
1 year postoperatively	89.82 ± 3.33	85.20 ± 3.80	<0.001

During the 1-year period, HHSs in both groups improved gradually and stabilized 1 year after the surgery. Before surgery, the HHSs in the C group were significantly lower than those in the D group (32.64 ± 9.11 vs. 46.32 ± 9.88, *P* < 0.05). The HHSs in the C group increased maximally between the time of operation and 3 months after the operation and exceeded those in the D group 3 months after the operation (71.71 ± 4.78 vs. 63.02 ± 6.17, *P* < 0.05), while the HHSs in group D showed increased maximally between 3 months and 6 months after operation. The HHS in the D group was significantly lower than that in the C group 1 year postoperatively (85.2 ± 3.8 vs. 89.8 ± 3.3, *P* < 0.05).

A simple linear regression was performed using time differences as independent variables. For the C group, the regression equation was HHS = 46.329 + (4.295 × time). The effect of time on the HHS is statistically significant (*P* < 0.001). Time accounted for 69.2% of the variation in the HHS, a moderate effect. For the D group, the regression equation was HHS = 51.760 + (3.082 × time). The effect of time on the HHS was statistically significant (*P* < 0.001). Time accounted for 73.1% of the variation in the HHS, a moderate effect. The HHS of the D group recovered more slowly than that of the C group.

### Health-related quality of life

EQ-5D_index_ scores were evaluated before surgery and 3 months, 6 months and 1 year postoperatively (Table 3). During the 1-year period, EQ-5D_index_ scores in both groups improved gradually. Before surgery, there were no differences in EQ-5D_index_ scores between the two groups (− 0.01 ± 0.02 vs. -0.009 ± 0.04, *P* > 0.05). The EQ-5D_index_ scores in the C group increased the most from the time of surgery to 3 months after surgery and exceeded those in the D group 3 months postoperatively (0.69 ± 0.05 vs. 0.55 ± 0.13, *P* < 0.05). The EQ-5D_index_ scores in the C group were still higher than those in the D group 6 months postoperatively (0.84 ± 0.10 vs. 0.75 ± 0.14, *P* < 0.05). There were no differences in EQ-5D_index_ scores between the two groups 1 year after surgery (0.95 ± 0.07 vs. 0.92 ± 0.07, *P* > 0.05).

**Table 3:** Mean EQ-5D_index_ scores before surgery and 3 months, 6 months and 1 year postoperatively in both groups

	C group	D group	*P* value
Before surgery	−0.01 ± 0.02	− 0.009 ± 0.04	0.073
3 months postoperatively	0.69 ± 0.05	0.55 ± 0.13	<0.001
6 months postoperatively	0.84 ± 0.10	0.75 ± 0.14	<0.001
1 year postoperatively	0.95 ± 0.07	0.92 ± 0.07	0.157

### The relationship between preoperative function and postoperative function at 1 year

Spearman correlation was used to determine the relationship between preoperative HHSs and postoperative HHSs at 1 year in the D group. The results showed that the HHS 1 year after the operation was related to the HHS before surgery, r_s_ = 0.521, *P* < 0.01.

### Patient satisfaction following surgery

Postoperative patient satisfaction was investigated 1 year after surgery. Three questions in the questionnaire concerning satisfaction with rehabilitation, pain treatment, and overall satisfaction (each on a scale of 25–100) were summed up, resulting in a total satisfaction score ranging from 25 to 100. The mean satisfaction score of the C group was 83.4 ± 8.2, while that of the D group was 91.8 ± 6.8. The difference was statistically significant (*P* < 0.05).

### Postoperative complications

In the D group, there was one case of hip dislocation 1 week postoperatively. The patient underwent manual reduction under general anesthesia, and the operated lower limb was fixed in a neutral position for 3 weeks. There was one patient with wound fat liquefaction and the wound healed after 1 week of dressing change. In the C group, one case of deep venous thrombus occurred, and the patient was treated with the anticoagulant drug low molecular heparin calcium for 5 weeks. One patient had a superficial infection in the wounds and was treated with antibiotics for 1 week. There was no nerve palsy or signs of prosthetic or periprosthetic fracture in any patients at the last follow-up.

### Causes of delay and comorbidities

The causes of delay in the D group were divided into four categories and are shown in Table 4. The comorbidities in the C group were as follows: type 2 diabetes, 5 patients; hypertension, 6 patients; type 2 diabetes and hypertension, 3 patients; cardiac insufficiency, 3 patients. The comorbidities in the C group were treated within 7 days before the operation.

**Table 4:** Causes of delay in the D group

Delayed causes	Patients (number)
Nonoperative treatments	20
Misdiagnosis	2
Missed diagnosis	3
Comorbidities	22
Recent myocardial infarction	7
Chronic obstructive pulmonary disease	4
Pulmonary embolism	3
Serious Cardiac insufficiency	8

## Discussion

The study results indicated that the hip function and quality of life of patients with femoral neck fracture whose surgery was delayed over 21 days recovered more slowly than those of pateints whose surgery was performed within 7 days. However, patients with surgery delayed over 21 days were more satisfied with the surgery. Although timely efficient surgery is well documented as the best management for elderly patients with femoral neck fracture [4, 5], some of these patients were unable to undergo early treatment due to various causes. Previous studies on femoral neck fracture focused on a relatively short delay time within 48 h and evaluated the trauma-related complications and mortality rate. However, in developing countries, some elderly patients may have extremely long-time surgery delays after a fracture [24, 25], and their hip function and quality of life were less concerned postoperatively.

### Hip function difference and muscle disuse

Many treatment guidelines on femoral neck fracture recommend that surgery should be performed as soon as possible after a femoral neck fracture. Most research concluded that patients who underwent early surgery recovered weight-bearing capacity and self-care ability earlier than those who did not [1, 13, 26]. Butler et al. reported that patients who underwent surgery over 12 h after admission functioned less well 6 weeks postoperatively [13]. However, this deleterious effect of delayed surgery on functional ability was not shown in Orosz’s study [27]. Their study found that having surgery within 24 h was not associated with improved function. In fact, patients in these studies all had acute fractures. In contrast, patients in our study all experienced extremely long delays before surgery.

Patients with delayed femoral neck fractures always suffer from shortening deformity and severe soft tissue contraction around the hip joint. In addition, elderly patients may experience severe muscle weakness due to disuse of the lower limbs, which requires soft-tissue release and scar removal during surgery. The lack of muscle strength affects functional recovery [28, 29], which may be the cause of the slower recovery of the HHSs in the D group. Thus, to facilitate functional recovery, measures should be taken to improve the lower limb muscles and strengthen the rehabilitation course. Furthermore, delay to surgery may have a direct effect on the incidence of subsequent dislocation of hip prostheses [30, 31]. This was thought to be linked to deterioration of patients’ physical condition and intraoperative soft tissue release. Long-term disuse of the hip resulting from delayed treatment can lead to serious hip osteoporosis, making the patient susceptible to intraoperative femoral fracture. Therefore, it is also important to select the appropriate prosthesis for this type of surgery.

Interestingly, in this study, we found that better HHSs before surgery indicated better HHSs 1 year postoperatively in the D group. For patients with delayed treatment of a femoral neck fracture, the reduction of hip motion resulted in disuse of the musculature around the hip, which may account for this phenomenon. We also observed that the patients in our study who had some mobility with the help of crutches had better postoperative hip function. This may be because hip motion contributes to maintaining hip muscle strength. A previous study showed that preoperative muscle strength affects functional recovery [28]. Therefore, it may be helpful for patients whose surgery has to be delayed to perform some hip motion, although it may cause hip pain.

In our study, HRQoL and the HHSs showed similar changes, and no long-term differences were seen. The HRQoL in the C group showed greater improvement after the operation. Many researchers have indicated that early surgery is associated with improved quality of life. Gjertsen et al. found that femoral neck fracture had a significant influence on HRQoL, and deterioration in the HRQoL was obvious even one year after the fracture [32]. Hüseyin Doruk et al. reported that elderly patients who underwent surgery within 5 days had a better quality of life at the 1-year follow-up than those who underwent surgery after 5 days [33]. Al-Ani et al. also found that early operation was associated with an improved ability to return to independent living [11]. Although our definition of ‘delay’ is generally longer than that of other studies, the results with regard to function and quality of life indicate that early surgery for delayed femoral neck fractures is still necessary.

We also determined patient satisfaction at the end of the follow-up. Patients in the D group had significantly higher satisfaction scores than those in the C group. We speculate that patients in the D group had suffered pain, reduced self-care ability and poor quality of life for a longer time than those in the C group. Thus, the improvements in personal feelings related to improved function and pain relief were more pronounced after the operation, which resulted in higher satisfaction scores although they had relatively lower hip function scores.

### Causes of delayed surgical treatment

In developing countries because of various causes, the time before surgical treatment may be several months for elderly patients with femoral neck fracture [34, 35]. Therefore, our study focused on a relatively long delay of intervention over 21 days. Twenty surgical delays in our study were due to patients’ preference for nonoperative treatment because of fear of surgery or belief in nonoperative treatment. Nonoperative treatment may be ineffective, and a significantly higher 30-day and 1-year mortality was shown in a recent meta-analysis of femoral neck fractures [36]. They chose surgery only after nonoperative treatment failed, which commonly resulted in a very long delay before surgical treatment. Therefore, adequate education should be given to patients and their guardians to aid decision-making. Accompanying comorbidity is also one of the main causes of surgical delay. A study of 571 patients with femoral neck fracture found that 123 (22.2%) patients had surgery over 48 h after arrival, of which, 78 (63%) were waiting for completion of a medical evaluation and 43 (35%) needed clinical stabilization [37]. Comorbidities should be optimized to avoid delays in surgery [5]. However, patients with femoral neck fracture who have serious cardiopulmonary comorbidities are at greater risk for surgery and some comorbidities (e.g. recent myocardial infarction and pulmonary embolism) may be surgical contraindications. Chinese doctors inform these patients about the high surgical risk. Some of them choose treatments to decrease the surgical risks. After a period of treatment for comorbidities and suffering of pain and inconvenience caused by fracture, they may finally decide to undergo surgery. Another cause for delay is misdiagnosis or missed diagnosis. Femoral neck fracture without displacement was sometimes not easy to see on anteroposterior hip radiograph. Then, with the movement of the hip, a nondisplaced fracture became displaced and was eventually noticed. Therefore, if the presumed fracture is not apparent on initial radiographs, hip computed tomography or a more specific check is necessary [5]. There were 3 patients with this condition in our study. Femoral neck fracture in elderly patients is a fragile fracture that could occur after a minor trauma that patients did not notice. Most of these patients had lumbar degeneration, which can also lead to the hip pain and reduced ground activities. In this condition, they would be treated as having lumbar degeneration without careful physical and imaging examination. There were 2 cases with this condition in our study.

This study has some limitations and strengths. First, this is a small study because the number of patients undergoing delayed treatment was relatively small. Additionally, the follow-up is limited. The strengths of this study are the reasonable matching and prospective nature of our study. For the recovery course of hip function after hip replacement, the larger extent of hip function recovery could be observed in the one-year term. More patients and longer follow-up should be used in further studies.

## Conclusion

Hip function in patients with femoral neck fracture and surgery delayed over 21 days recovered slower than those with surgery performed within 7 days. However, those with longer delays were more satisfied with the surgery. Moderate hip movement to ameliorate lower limb muscle atrophy was recommended for patients facing a temporary inability to undergo surgery. More importantly, some measures should be taken to avoid delayed treatment in elderly patients with femoral neck fracture.

## Data Availability

The dataset used and analyzed during the current study, which are maintained by designated researchers, are available through the corresponding author upon reasonable request.

## Abbreviations

HHS: Harris hip score
HRQoL: Health-related quality of life
